# Polypeptide Self-Assembled Nanoparticles as Delivery Systems for Polymyxins B and E

**DOI:** 10.3390/pharmaceutics12090868

**Published:** 2020-09-11

**Authors:** Dmitrii Iudin, Natalia Zashikhina, Elena Demyanova, Viktor Korzhikov-Vlakh, Elena Shcherbakova, Roman Boroznjak, Irina Tarasenko, Natalya Zakharova, Antonina Lavrentieva, Yury Skorik, Evgenia Korzhikova-Vlakh

**Affiliations:** 1 Institute of Macromolecular Compounds, Russian Academy of Sciences, 199004 St. Petersburg, Russia; dmitriy-yudin97@mail.ru (D.I.); nzashihina@bk.ru (N.Z.); itarasenko@list.ru (I.T.); na_zar@inbox.ru (N.Z.); yury_skorik@mail.ru (Y.S.); 2 Saint-Petersburg State University, Institute of Chemistry, 198584 St. Petersburg, Russia; v_korzhikov@mail.ru; 3 State Research Institute of Highly Pure Biopreparations, Federal Medical-Biological Agency, 197110 St. Petersburg, Russia; lenna_22@mail.ru (E.D.); elenka.shcherbakova@ya.ru (E.S.); 4 Department of Materials and Environmental Technology, Tallinn University of Technology, 19086 Tallinn, Estonia; prionolog@gmail.com; 5 Institute of Technical Chemistry, Gottfried-Wilhelm-Leibniz University of Hannover, 30167 Hannover, Germany; lavrentieva@iftc.uni-hannover.de

**Keywords:** peptide antibiotics, polymyxins, drug delivery systems, polypeptide nanoparticles, polymyxin loading and release, minimal inhibitory concentration

## Abstract

Polymyxins are peptide antibiotics that are highly efficient against many multidrug resistant pathogens. However, the poor stability of polymyxins in the bloodstream requires the administration of high drug doses that, in turn, can lead to polymyxin toxicity. Consequently, different delivery systems have been considered for polymyxins to overcome these obstacles. In this work, we report the development of polymyxin delivery systems based on nanoparticles obtained from the self-assembly of amphiphilic random poly(l-glutamic acid-*co*-d-phenylalanine). These P(Glu-*co*-dPhe) nanoparticles were characterized in terms of their size, surface charge, stability, cytotoxicity, and uptake by macrophages. The encapsulation efficiency and drug loading into P(Glu-*co*-dPhe) nanoparticles were determined for both polymyxin B and E. The release kinetics of polymyxins B and E from nanoformulations was studied and compared in buffer solution and human blood plasma. The release mechanisms were analyzed using a number of mathematical models. The minimal inhibitory concentrations of the nanoformulations were established and compared with those determined for the free antibiotics.

## 1. Introduction

**Figure 1:**
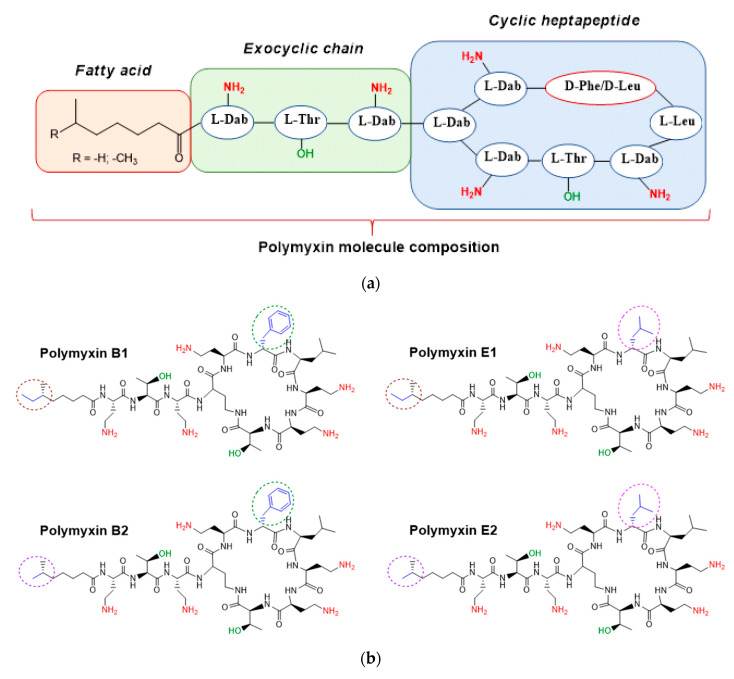
General polymyxin molecule composition (**a**) and structures of polymyxins B and E (**b**).

Polymyxins are cyclic cationic peptides consisting of 10 amino acid residues. This group of antibiotics, which includes polymyxins A, B, C, D, E, and M, is biosynthesized by various species of *Paenibacillus polymyxa* [1,2]. Among the polymyxins, polymyxin B and polymyxin E (colistin) are used in clinical practice. These two polymyxins differ by only one amino acid in their structure: a leucine in polymyxin E is replaced by a phenylalanine in polymyxin B. Both polymyxins B and E exist as mixtures of closely related lipopeptides that differ in the structure of the N-terminal fatty acyl group [3]. Commercial preparations of both polymyxins (B and E) contain two different cyclic forms of the peptides in ratios that can vary from batch to batch. Polymyxin B preparations contain polymyxin B1 and B2, whereas polymyxin E preparations contain polymyxin E1 and E2 (or colistin A and colistin B) (Figure 1).

Polymyxins were discovered in the middle of the 20th century as effective treatments for Gram-negative bacterial infections. Polymyxins carry a strong positive charge, so they bind to the negatively charged bacterial cell envelope, thereby disrupting the membrane and causing leakage of the bacterial cell contents [4,5]. Polymyxins are mainly active against Gram-negative pathogens, such as *Escherichia coli*, *Klebsiella* spp., *Enterobacter* spp., *Pseudomonas aeruginosa,* and *Acinetobacter* spp. [6]. However, the low stability of polymyxins in the bloodstream requires the administration of high doses of the drug to reach the therapeutic threshold. This practice has led to the emergence of negative side-effects, such as neuro-, hepato- and nephrotoxicity [7,8]. Currently, the nephrotoxicity is the major factor limiting the wide applications of polymyxins in clinical practice [9,10]. The development of novel and less toxic antibiotics toward the end of 20th century favored the progressive abandonment of polymyxins as therapeutics. However, in the past few years, the shortage of new therapeutic options due to the appearance of resistant Gram-negative bacteria has led to a re-emergence of polymyxins in clinical practice [11]. Currently, polymyxins are still very active against many multidrug resistant pathogens, e.g., *P. aeruginosa,* and now represent the treatment of choice when other antibiotics are contraindicated due to antimicrobial resistance [12,13]. 

The application of appropriate nanomedicine systems can (i) improve drug delivery, (ii) diminish the dose (and, in turn, the toxicity), (iii) provide prolonged release of the drugs, and (iv) elicit a longer duration of action, combined with reduced frequency of administration [14]. A number of publications has been devoted to the encapsulation of antimicrobial peptide drugs into polymer systems. A particular focus for these drugs has been the development of delivery systems based on nanoparticles (NPs) of poly(lactide-*co*-glycolide) [15,16] and chitosan [17], as well as different poly(methacrylic acid) microgels [18]. Summaries of different delivery systems available for antibacterial peptide drugs have been presented in some recent reviews [19,20,21]. 

Delivery systems for polymyxins need to take into account the chemical nature of these drugs. Polymyxins contain five primary aliphatic amino groups that allow their successful entrapment by anionic molecules and polyanions. Besides the positive charge, the presence of a lipophilic tail, as well as the hydrophobic amino acids in the polymyxin peptide structure, allows the use of both amphiphilic and hydrophobic low-molecular compounds and polymers for drug delivery. Therefore, the delivery systems considered for polymyxins include anionic liposomes [22,23,24,25], lipid NPs [26,27], and different polymer systems [28,29,30]. The polymers include hydrophobic poly(butyl cyanoacrylate)-based NPs [29], poly(l-lactide)/halloysite nanotube nanofiber mats for wound treatment [30], and hybrid systems based on alginate-cross-linked polymyxin B loaded into lipid NPs [28]. In addition, the development of polymyxin delivery systems based on negatively charged polymer micro- and nanoparticles have also been reported. For instance, Coppi et al. developed a set of microparticles consisting of alginate and chitosan polyplexes for oral administration of polymyxin B [31,32]. Similarly, Liu et al. prepared NPs by complexation of colistin with poly(glutamic acid) and further stabilized this delivery system with 1,2-dimyristoyl-sn-glycero-3-phosphoethanolamine-*N*-(methoxy (polyethylene glycol)-2000) [33]. 

In the present work, we have proposed the application of random polypeptides composed of negatively charged and hydrophobic amino acids. These polypeptides are capable of polymyxin entrapment through both electrostatic and hydrophobic interactions. The characteristics of NPs and their morphology, biological properties, encapsulation efficiency, drug loading, and release kinetics have been carefully investigated. The antimicrobial efficacy was determined by exposing *P. aeruginosa* to the polymer-based polymyxins or to free polymyxins and then measuring and comparing the respective antibacterial activity.

## 2. Materials and Methods 

### 2.1. Chemicals and Supplements 

Polymyxin B (sulfate) and polymyxin E (colistin sulfate) were procured from Fluka (Munich, Germany) and BetaPharma (Wujiang, Shanghai, China), respectively. The γ-benzyl-l-glutamic acid (Glu(OBzl), d-phenylalanine (d-Phe), α-pinene, *n*-hexylamine, triphosgene, trifluoromethanesulfonic acid (TFMSA), and trifluoroacetic acid (TFA) were obtained from Sigma-Aldrich (Darmstadt, Germany) and used as received. All organic solvents used for synthesis were purchased from Vecton Ltd. (St. Petersburg, Russia) and distilled and dried before use according to standard protocols. ACS reagent grade salts used for buffer preparation were also purchased from Vecton Ltd. (St. Petersburg, Russia). The buffer solutions were prepared by dissolving salts in deionized water and further purified by filtration through a 0.45 μm Milex membrane microfilter (Millipore Merck, Darmstadt, Germany). Amicon membrane tubes used for ultrafiltration (MWCO 3000) were also Merck products (Darmstadt, Germany). Spectra/Pore^®^ (MWCO: 1000) dialysis bags were purchased from Spectra (Rancho Dominguez, CA, USA). Poly(methyl methacrylate) (PMMA) standards (*M_w_* = 17,000−250,000; *Đ* ≤ 1.14) purchased from Supelco (Bellefonte, PA, USA) were used for size exclusion chromatography (SEC) column calibration. Mouse “self-peptide” [34] (GNYTCEVTELTREGETIIELK) was synthesized in IMC RAS (St. Petersburg, Russia) via conventional solid-phase peptide synthesis using BOC-strategy. The poly(lactic acid) (PLA) and poly(lactic acid)-*b*-poly(ethylene glycol)-5000 (PLA-*b*-PEG) NPs used for comparison in this work were kindly donated by Dr. Ekaterina Sinitsyna from the Institute of Macromolecular Compounds of the Russian Academy of Sciences (St. Petersburg, Russia). 

### 2.2. Cell Lines and Bacterial Strain

Human kidney embryonic cells (HEK 293, CLS Cell Lines Service GmbH, Germany), human bronchial epithelial cells (BEAS-2B, ATCC^®^ CRL-9609™, USA), and mouse BALB/c monocyte macrophages (J774A.1, CLS Cell Lines Service GmbH, Germany), were used for the study of NPs cytotoxicity and NPs uptake by macrophages. The test culture for minimum inhibitory concentration (MIC) evaluation was *P. aeruginosa ATCC 27,853* from the collection of microorganisms of the State Research Institute of Highly Pure Biopreparations (St. Petersburg, Russia).

### 2.3. Molecular Docking 

Molecular docking procedures were conducted using AutoDock 4.2.6 (The Scripps Research Institute, La Jolla, CA, USA) and were applied to selected center atoms and the whole colistin (receptor) rigid grid box. The crystal structure of colistin (5L3G:C) deposited in the Protein Data Bank was processed within Spartan to generate a structure compatible with the docking limitations. For rigid docking, a rigid receptor grid, defined by an inner box of 80 × 80 × 80 Å^3^, was generated for colistin with the center at 20.568, −0.871, and 3.311 Å. Poly(glutamic acid) and poly(aspartic acid) were limited by 5 units due to docking software limitations. For this reason, penta-glutamate (Glu_5_), penta-aspartate (Asp_5_), and (Glu_2_PheGlu_2_) structures were generated in Spartan. The images of the molecular docking simulations for polymyxin E and pentapeptides are presented in Figure S1. All ligands were adopted for AutoDock 4.2.6 software by selection of residue numbers before 32 torsions. The ligands and the colistin were converted into the AutoDock tools format (into .pdbqt files). The initial files for the grid-box (.gpf) and docking algorithm (.dpf) were prepared with AutoDock tools and processed with AutoDock 4.2.6 software. The candidate ligands were scored into the colistin as flexible ligands with selection spacing 0.375, smooth 0.5, and a genetic algorithm (4.2) with a population of 150.

### 2.4. Synthesis and Characterization of Polypeptides 

Amphiphilic random polypeptides were synthesized using the ring-opening polymerization (ROP) of the amino acid *N*-carboxyanhydrides (NCA). NCA monomers were synthesized as described elsewhere [35]. Tetrahydrofuran (THF) was used as a solvent for Glu(OBzl) and d-Phe NCA synthesis. The products were recrystallized three times from ethyl acetate/*n*-hexane (yields: Glu(OBzl) NCA, 73%; d-Phe NCA, 45%). The structure and purity of the NCAs obtained were verified by ^1^H NMR at 25 °C in CDCl_3_. The spectra were recorded at 298 K using a Bruker 400 MHz Avance instrument (Karlsruhe, Germany). Glu(OBzl) NCA: 2.05–2.39 (m, 2H), 2.63 (t, 2H), 4.39 (t, 1H), 5.17 (s, 2H), 6.40 (br. s., 1H), 7.28–7.39 (m, 5H); d-Phe NCA: 2.94–3.35 (m, 2H), 4.55 (m, 1H), 6.12 (s, 1H), 7.19–7.41 (m, 5H).

NCAs copolymerization was carried out in freshly distilled THF. Weighed portions of the monomers were separately dissolved in THF according to a predetermined ratio, and a final solution of NCAs (M1 + M2) with a concentration of 4 wt % was prepared. A solution of *n*-hexylamine (initiator) in THF was then added to the reaction mixture to achieve a ratio of [M1 + M2]/[I] = 100. This polymerization mixture was incubated at 25 °C with stirring. After 48 h, the resulting copolymers were precipitated in diethyl ether. The precipitate was separated by centrifugation at 10,000 rpm for 5–7 min, washed three times with diethyl ether, and then dried under vacuum. All obtained copolymers were analyzed by SEC using a tandem of two Agilent PLgel MIXED-D columns (7.5 mm × 300 mm, 5 μm) (USA) and a Shimadzu LC-20 Prominence system supplied with a refractometric RID 10-A detector (Kyoto, Japan). The analysis was performed in a 0.1 M solution of LiBr in dimethyl formamide (DMF) as an eluent, at a flow rate of the mobile phase of 1 mL/min and a temperature of 40 °C. Molecular weight characteristics were calculated using GPC LC Solutions software (Shimadzu, Kyoto, Japan) and the calibration curve was plotted using PMMA standards. SEC traces are presented in Figure S2.

The molecular weights for macromolecules were measured by static light scattering (SLS) method in dimethyl sulfoxide at 21 °C. Light scattering was determined on a Photocor Complex unit (Photocor Instruments, Moscow, Russia); a Photocor-DL diode laser served as a light source (power of 5–30 mW, wavelength λ = 659.1 nm). The instrument was calibrated with benzene (*R_V_* = 2.32 × 10^−5^ cm^−1^). The correlation function of the scattered light intensity was obtained from the use of a Photocor-PC2 correlator with 288 channels and was processed using DynalS software (Photocor Instruments, Moscow, Russia). In these solutions, no asymmetry of light scattering occurred; thus, the *M*_w_ of the copolymers was determined by the Debay method (Table S1). The refractive index increments were measured by an RA-620 refractometer (KEM, Kyoto, Japan).

The removal of the benzyl protective group from γ-carboxyl of glutamic acid was carried out with the use of TFMSA/TFA mixture in a ratio of 1/10 at 25 °C for 3 h. The deprotected copolymers were then precipitated in diethyl ether, and the precipitate was separated by centrifugation at 11,000 rpm for 10 min. The resulting precipitate was washed three times with diethyl ether and dried. NMR spectra recorded for protected and deprotected polypeptide are given in Figure S3.

The composition of the copolymers was determined by the quantitative HPLC (LC-20 Shimadzu system with refractometric RID-20A detector, Shimadzu, Kyoto, Japan) analysis of free amino acids. For this, the copolymer samples were hydrolyzed to free amino acids and analyzed as described in [36]. 

### 2.5. Formation and Characterization of Nanoparticles 

Polymer particles were formed during gradient phase inversion (dialysis). For this, the dried polypeptide was dissolved in DMF, dialyzed against DMF/water and then against water using membrane bags with MWCO 1000. The milky suspension was then frozen and freeze-dried. The NPs were redispersed by placing a weighed amount of polymer particles into medium of interest (water or buffer solution) and performing a short ultrasonication (30 s) at 20% power with a SONOPULS HD2070 ultrasonic homogenizer (Bandelin, Berlin, Germany).

The hydrodynamic diameter (*D_H_*) of the NPs, their polydispersity index (*PDI*), and electrokinetic potential (ζ-potential) were measured by dynamic light scattering (DLS) and electrophoretic light scattering using a Zetasizer Nano ZS instrument (Malvern Instruments, UK). The measurements were conducted on 1 mL suspensions of NPs (0.3 mg/mL). The light scattering measurements were performed in deionized water, DMEM, 0.01 M phosphate buffer (PB), and 0.01 M phosphate buffered saline (PBS), pH 7.4, whereas the ζ-potentials were determined in deionized water.

The morphology of the obtained NPs was studied by transmission electron microscopy (TEM) (Jeol JEM-2100 HC, Tokyo, Japan). Samples were prepared for analysis by placing 8 μL of a suspension of NPs onto a copper grid with a carbon coating of 300 mesh and drying in air. The grid was then immersed in a 3% solution of uranyl acetate for 1 min, washed gently with pure water, and dried for 30 min before the measurement. The study was carried out at an accelerating voltage of 160 kV. Three TEM images were analyzed by ImageJ software (Madison, VI, USA) to calculate the average NP size.

The critical micelle concentration (CMC) was determined by conductometry using a SevenCompact Cond meter S230 conductometer (Mettler Toledo, Columbus, OH, USA). The measurements were performed in a range of concentrations from 2.8 to 8.9 µg/mL at 25 °C. The CMC was determined as the intersection point of two linear sections of the conductivity versus polymer concentration plots (Figure S4).

The stability of the NPs was verified in both 0.01 M PB (pH 7.4) and DMEM-FCS (Dulbecco’s Modified Eagle Medium containing Fetal Calf Serum) culture medium. A 0.3 mg/mL NP suspension was incubated at 37 °C with stirring for 21 days. The sample *D_H_* and *PDI* were monitored by DLS. 

The modification of the surface of P(Glu-*co*-dPhe) NPs with “self”-peptide was carried out using the procedure developed earlier for other peptides [37].

### 2.6. Analysis of Commercial Drug Preparations 

The composition of commercial preparations was determined by LC-MS analysis. Chromatographic separation was performed using an Elute UHPLC system (Bruker Daltonics GmbH, Bremen, Germany) equipped with a Millipore Chromolith Performance/PR-18e, C18 analytical column (100 mm × 2 mm, Merck, Darmstadt, Germany) with a Chromolith RP-18 endcapped 5-3 guard cartridge (Merck, Darmstadt, Germany), operated at a flow rate of 300 μL/min. Mobile phases were as follows: A, a 100:1:0.03 (*v/v/v*) mixture of water/acetonitrile/trifluoroacetic acid and B, acetonitrile. The elution gradient was as follows: 0–1 min linear gradient of B from 20% to 40%; 1–1.1 min, linear gradient of B from 40% to 90%; 1.1–2 min, isocratic elution with 90% B; 2–2.1 min, linear gradient of B from 90% to 20%; 2.1–4 min, isocratic elution with 20% B. The total run time was 4 min, the injection volume was 1 μL, and the polymyxin concentration was 10 μg/mL.

Mass spectra were obtained with a Maxis Impact Q-TOF mass spectrometer (Bruker Daltonics GmbH, Bremen, Germany) equipped with an electrospray ionization (ESI) source (Bruker Daltonics GmbH, Bremen, Germany) and operated in positive ionization mode. Mass calibration was carried out with 0.1 M sodium formate in a water: methanol (50:50, *v/v*) solution. Acquisition parameters were electrospray voltage of 4.5 kV and scan range for MS was 50–1000 *m/z*. The mass spectra were analyzed using Data Analysis and TASQ software (Bruker Daltonics GmbH, Bremen, Germany). 

### 2.7. Drug Loading

The encapsulation of polymyxins was carried out by adding 100 µL of antibiotic solution at different concentrations to 900 µL of a suspension of freshly redispersed NPs (in 0.01 M PB, pH 7.4). The final NPs concentration was 1 or 2 mg/mL. The resulting mixture was thoroughly vortexed and left at 4 °C for 20 h. The nonencapsulated drug was removed from the solution by ultrafiltration using tubes supplied with MWCO 3000 membranes. The filtrates were collected, lyophilized, dissolved in eluent A, and analyzed by HPLC. The stationary phase was a cation-exchange CIM-SO_3_ disk (12 × 3 mm) from BIA Separations (Slovenia). The eluents were the following: A, 0.005 M sodium phosphate buffer, pH 7; B, 0.005 M sodium phosphate buffer, pH 7, containing 2 M NaCl. The analysis was carried out using the following binary gradient: 0–2 min, 100% A; 2–8 min, 0 to 100% B; 8–12 min, 100% B. The flow rate of the mobile phase was 1 mL/min and the eluted compounds were detected at a wavelength of 215 nm. The elution time was 6.0–6.2 min for polymyxin E and 7.8–8.0 min for polymyxin B. The concentration in analyzed solutions was calculated from the peak area and its correlation with calibration plot built for standard polymyxin solutions (0.05–8 mg/mL). The loading capacity (*LC*) and encapsulation efficacy (*EE*) were calculated according to the following equations:

*LC* = (*m_i_* − *m_s_*)/*m_NP_* (1)

*EE* = (*m_i_ − m_s_*)/*m_i_* × 100% (2)

where *m_i_* is the initial polymyxin mass (mg), *m_s_* is the mass of nonencapsulated polymyxin in solution (mg), and *m_NP_* is the mass of the NPs (mg).

### 2.8. Release Study

The release of polymyxins from the NPs was studied in 0.01 M PBS, pH 7.4, and in human blood plasma. A 1.0 mL volume of NP colloids (1.0 mg/mL) containing 380 ± 16 µg of polymyxin B/mg of NPs or 322 ± 10 µg of polymyxin E/mg of NPs was incubated at 37 °C for 3 days. At predetermined time intervals, the free peptide was separated from the NPs by ultracentrifugation using a microtube fitted with a 3000 MWCO membrane. A corresponding aliquot of buffer was added to the NPs and the solution was filtered again. The procedure was repeated three times. The filtrates were combined, lyophilized, and then analyzed by HPLC as described above. 

Several mathematical models were applied for mechanism analysis of polymyxin release from the NPs. The linearization of the release data was evaluated according to zero-order, first-order, Higuchi, Hixson-Crowell, Korsmeyer-Peppas, Baker-Lonsdale, Hopfenberg, Weibull, Gompertz, and Peppas-Sahlin models using the DDSolver add-in for Microsoft Excel (freely available software, which was developed by Zhang Yong and colleagues from China Pharmaceutical University [38]). The relevant correlation coefficients were analyzed to define the model, as these are better for description of release in different environments. 

### 2.9. Cytotoxicity Study and Uptake by Macrophages

The cytotoxicity study was carried out using HEK 293 and BEAS-2B cell lines. Toxicity was assessed using the Cell Titer-Blue (CTB) reagent test. This test is based on the ability of living cells to convert resazurin to the fluorescent product resorufin. The amount of detected resorufin fluorescence (λ_ex._ = 545 nm, λ_em._ = 590 nm) is proportional to the number of viable cells. For the experiment, 8 × 10^3^ cells were seeded into each well of a 96-well plate in 100 μL of culture medium containing solutions of basal medium, fetal bovine serum (FBS), penicillin, and streptomycin. The cells were cultured for 24 h in an incubator at 37 °C in a humidified 5% CO_2_ atmosphere. The culture medium was then aspirated, and 200 μL of culture medium containing NPs of various concentrations (*n* = 9), polymyxin B, or polymyxin B nanoformulation (*n* = 4) was added. In the case of encapsulated polymyxin B, the content of polymyxin B in the stock nanoformulation was 300 μg/mg of NPs. The cells were incubated for 24 or 72 h, then the medium was removed and 100 μL of CTB reagent solution (10% stock solution in basal medium) was added to each well. The cells were incubated in the CO_2_ incubator for 1–3 h at 37 °C, and then, the fluorescence of the solution was measured. The data obtained were normalized as a percentage of the control.

The uptake of particles by macrophages was determined by flow cytometry on a BD Accuri C6 instrument (Becton Dickinson, Franklin Lakes, NJ, USA). A 2 mL volume containing 3 × 10^6^ cells in DMEM culture medium was placed into each well of 6-well plates and cultivated for 24 h in a humidified 5% CO_2_ environment at 37 °C until 70–80% confluence was reached. The medium was then removed and replaced with fresh medium (1 mL) containing NPs labeled with Cy5. The NP concentration was 50 µg/mL; the Cy5 amount was 3 µg/mg of NPs. The plates were incubated in 5% CO_2_ at 37 °C for different times (0–24 h). The cells were then washed three times with warm PBS, detached with a cell lifter, and resuspended in 500 µL PBS. The fluorescence signals were then measured by flow cytometry on a BD Accuri C6 instrument with a 488 nm argon-ion laser. Fluorescence signals were collected using a 670 LP band-pass filter. At least 30,000 events per sample were analyzed. Only viable cells were used in the analysis. 

### 2.10. Antibacterial Activity 

The MIC of antibiotics was determined using the microdilution method in Mueller-Hinton broth (HiMedia, India). An overnight (18 h) culture of *P. aeruginosa ATCC 27,853* was used. A 125 μL volume of Mueller-Hinton broth was added to 96-well sterile flat-bottomed plates. Two-fold concentrations of colistin (125 μL) were then added to the wells of the first column, resuspended, and transferred to the next column to make repeated antibiotic dilutions to the penultimate column on the plate. The final series of antibiotic dilutions ranged from 64 to 0.25 μg/mL.

Absorbance of an overnight *P. aeruginosa* suspension in Mueller-Hinton broth was measured by the spectrophotometer UVmini-1240 (Shimadzu) at a wavelength of 630 nm and a cell concentration 1 × 10^7^ CFU/mL was prepared by serial dilutions (1:10) in Muller-Hinton broth. Negative controls (sterility of broth), positive controls (culture growth without antibiotic), and NPs controls were also present on the plate. The MIC was determined both visually by turbidity assessment and calculated based on absorbance values. The growth of *P. aeruginosa* was compared with the presence of antibiotic in culture growth and without it. After 18 h incubation at 37 °C, the absorbance in the wells at 630 nm was measured using an ELx808 BioTek Microplate Reader (Winooski, VT, USA). Each sample was tested thrice in three independent series (*n* = 9). The graphs were plotted with the use of mean value ± SD.

### 2.11. Statistics

The statistical significance among the groups was evaluated using the one-way analysis of variants in Excel with the XLSTAT. The data are presented as mean ± SD. Number of repeats for the same sample was varied from 4 to 9 (see above). The results counted as a significantly different when *p* ≤ 0.05. 

## 3. Results and Discussion 

### 3.1. Synthesis of Polymers

Polymyxins are positively charged and have hydrophobic moieties in their structures. Therefore, negatively charged amphiphilic random polypeptides were selected as potential polymer systems for loading of polymyxins in this study. The proposed polymers can undergo both electrostatic and hydrophobic interactions with the polymyxin drugs of interest. At the same time, their amphiphilic nature is a driving force for self-assembly of these polymers, and drug entrapment can occur during this self-assembly process. 

In natural polypeptides, a negative charge is provided by presence of glutamic or aspartic acids. The choice of negatively charged amino acid for polymer synthesis was made prior to the experiment by calculating the energetically favorable binding strength between the drug and corresponding amino acids using a molecular docking approach. Molecular docking is a key tool for predicting the predominant binding mode(s) of a ligand (a peptide or any other molecule) with a protein/peptide. The binding strength prediction was limited to poly(glutamic acid) and poly(aspartic acid) peptides of 5 units due to docking software limitations. Colistin was used as the target polymyxin antibiotic. A stronger complex formed between colistin and a glutamic acid oligomer (binding energy 10.1 kJ/mol) than between colistin and an aspartic acid oligomer (binding energy 7.1 kJ/mol). Thus, glutamic acid was selected for the synthesis of polypeptide delivery systems.

The selected hydrophobic amino acid was phenylalanine. The stability of polypeptides against enzymatic hydrolysis was improved using d-phenylalanine instead of the l-isomer. The P(Glu-*co*-dPhe) polypeptides were synthesized using *n*-hexylamine-initiated ring-opening polymerization of α-amino acid *N*-carboxyanhydrides (NCA). The polypeptides were characterized by SEC, SLS, and HPLC analysis to determine their molecular weight (SEC and SLS), dispersity (SEC), and composition (HPLC). The composition of polypeptides was determined by hydrolyzing them to free amino acids and determining the quantity by HPLC amino acid analysis. 

Table 1 summarizes the characteristics of the synthesized polypeptides. As seen, both samples have similar molecular weights and low dispersity. The determined composition was close to the initial monomer ratio. For our further experiments, we selected sample 2, which had a lower content of the hydrophobic amino acid. 

### 3.2. Formation and Characterization of Nanoparticles

The self-assembly of amphiphilic polypeptides was carried out by gradient phase inversion (dialysis) because of its simplicity and suitability for preparing self-assembled nanosystems [32,33,34]. The obtained suspensions were freeze-dried for storage. Before use, weighed samples were redispersed in 0.01 M PB (pH 7.4) or in water, depending on the goal, by short-term ultrasonication (30 s). The *D_H_*, *PDI*, and ζ-potential values of NPs were measured using DLS and electrophoretic light scattering. The close molecular weights and compositions for both synthesized polypeptides would predict a similar trend in the formation of NPs, and the self-assembled P(Glu-*co*-dPhe) NPs had close *D_H_*, *PDI,* and ζ-potential values (Table 2). The CMC of Sample 2, determined by conductometry [39], was found by a linear fitting of the two data subsets and calculating the concentration at their intersection (Figure S4). The determined value was close [40] or even lower [41] that of other known amphiphilic random copolymers.

**Figure 2:**
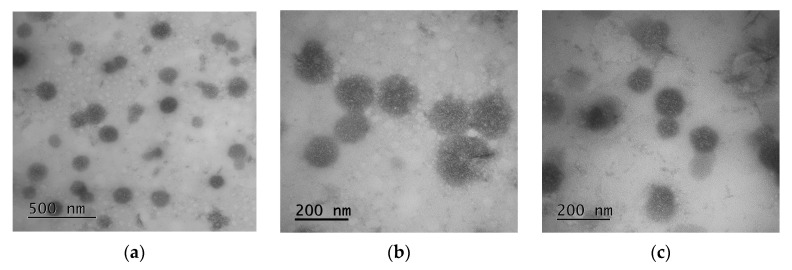
TEM images of P(Glu-*co*-dPhe) nanoparticles (staining with uranyl acetate): (**a**,**b**) empty nanoparticles and (**c**) polymyxin B-loaded nanoparticles.

The morphology of NPs was evaluated by TEM and with uranyl acetate staining (Figure 2). The TEM images indicated that the NPs were spherical, with an average diameter range of 121 ± 27 nm (Figure 2a,b). The smaller average diameter of NPs determined from the TEM images can reflect the loss of water as the sample dried on the TEM grid. This is a well-known feature of soft self-assembled nanomaterials [42,43].

**Figure 3:**
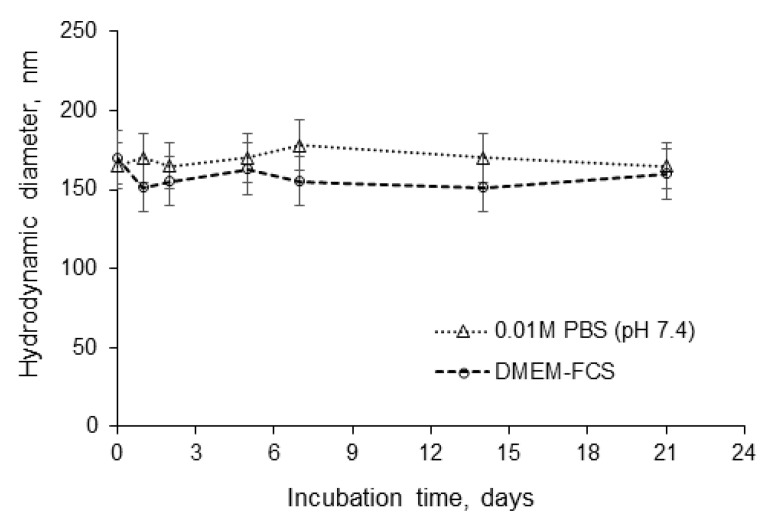
Stability of nanoparticles against aggregation in a model buffer solution and in DMEM-FCS culture medium during incubation at 37 °C for 3 weeks.

The high absolute value of ζ-potential was premised on the assumption of a potentially high stability of NPs in the dispersion against aggregation [44]. Indeed, no aggregation or sedimentation was detected for the P(Glu-*co*-dPhe) NPs (sample 2) when incubated in 0.01 M PBS (pH 7.4) or in DMEM-FCS culture medium for 3 weeks at 37 °C (Figure 3). Moreover, as it was expected, the introduction of d-amino acid into polypeptide structure improved its stability towards enzymatic degradation (Figure S5). 

### 3.3. Biological Evaluation

**Figure 4:**
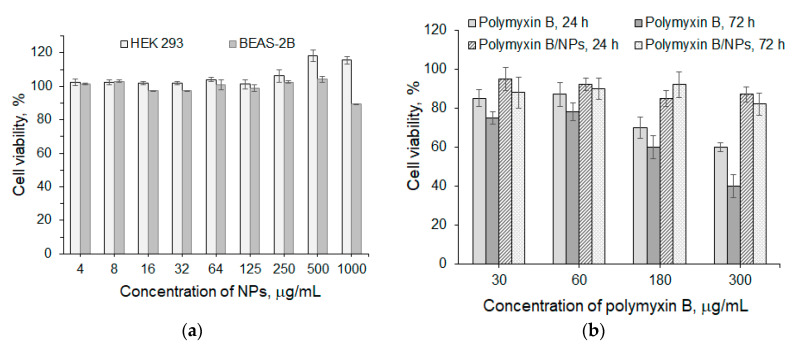
Viability of HEK 293 and BEAS-2B cell lines incubated in the presence of P(Glu-*co*-dPhe) for 72 h (**a**) and HEK 293 in the presence of polymyxin B loaded into P(Glu-*co*-dPhe) NPs and free polymyxin B for 24 and 72 h (**b**). The content of polymyxin B in the stock nanoformulation was 300 μg/mg of NPs. The data are given as mean ± SD. The differences between groups were statistically significant ((**a**) *p* < 0.001; (**b**) *p* < 0.05)).

The in vitro cytotoxicity of NPs was evaluated in HEK 293 and BEAS-2B cell lines. The cell viability was determined after 72 h using the CTB Assay based on the reduction of blue resazurin to purple resorufin by metabolically active cells. No cytotoxicity was detected at concentrations up to 1.0 mg/mL in either cell line (Figure 4a). In order to evaluate the potential toxicity of the developed polymyxin delivery systems towards kidney cells, we additionally tested polymyxin B loaded into P(Glu-*co*-dPhe) NPs in HEK 293 cells (Figure 4b). In contrast to free polymyxin B, P(Glu-*co*-dPhe)-based polymyxin B delivery system demonstrated a viability of HEK 293 cells higher than 80% after both 24 and 72 h of coincubation. 

Another important property of NPs that is under consideration as delivery systems is their capacity for uptake by macrophages. The NPs are foreign objects, so they are targeted for elimination from the organism through phagocytosis. Negatively charged NPs are known to be less prone to blood clearance than the positively charged ones [45]. Indeed, NPs are often surface modified with PEG to prevent their fast capture by macrophages. Taking this into account, PLA and PLA-*b*-PEG, which are widely used as delivery systems, were selected as benchmarks for comparison with the developed P(Glu-*co*-dPhe) NPs. Characteristics of used PLA and PLA-*b*-PEG NPs can be found in Table S2.

**Figure 5:**
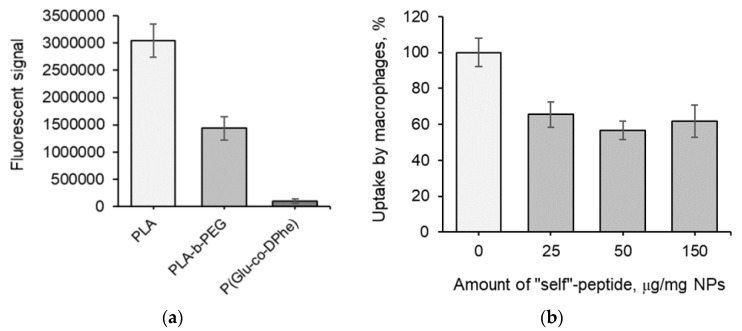
Uptake of different nanoparticles by macrophages (**a**) and effect of P(Glu-*co*-dPhe) modification with “self”-peptide on uptake by macrophages (**b**). *Conditions:* J774A.1 cell line; flow cytometry; NP concentration was 50 μg/mL, amount of Cy5 was 3 μg/mg NPs; 6 h. The data are given as mean ± SD. The differences between groups were statistically significant (*p* < 0.05).

Flow cytometry was applied for quantitative determination of NP uptake by macrophages. The internalization was studied by incubating J774A.1 cell line with Cy5-labeled NPs for 6 h. Figure 5A shows that the highest uptake by macrophages was observed for PLA NPs. The introduction of PEG into the polymer structure favored a slowdown of the NP uptake. When compared with control NPs, the P(Glu-*co*-dPhe) NPs demonstrated the lowest uptake by macrophages. Taking into account both the low cytotoxicity and the low macrophage uptake, the NPs obtained can be considered as prospective systems for polymyxin delivery. 

Recently, a so-called “self”-peptide, a fragment of the CD47 cell receptor, was discovered to serve as a signaling molecule that regulates phagocytosis [34]. In our previous work, we observed a decreased phagocytosis of PLA NPs modified with the “self”-peptide [46]. This concept was confirmed for the polypeptide systems by modifying the P(Glu-*co*-dPhe) NPs with a fluorescent dye and further modifying a fraction of them with the “self”-peptide. 

Initially, we studied the uptake rate for neat and modified NPs and found no increase in the amount of phagocytosed NPs after 6 h (Figure S6). Figure 5B shows the effect of varying the amount of “self”-peptide conjugated to the NPs (25, 50, and 150 µg/mg of NPs). The modification of NPs with the “self”-peptide diminished their uptake by macrophages by 35–43% after 6 h when compared with nonmodified control NPs. However, no reliable difference was observed for the NPs modified with different amounts of “self”-peptide. Therefore, the optimal range of “self”-peptide loading can be considered to fall between 25 and 50 µg/mg of NPs. In general, the suppression of NP uptake by macrophages can be quite useful in situations that require intravenous injection and a prolonged circulation of a drug delivery system in the bloodstream.

### 3.4. Loading of Polymyxins into Nanoparticles 

We further evaluated the precise composition of potential commercial preparations, as this might affect the loading and release properties. HPLC-MS analysis of polymyxin B and E samples (Table 3) revealed that polymyxin E was enriched in the E2 subtype that is acylated with a less hydrophobic 6-methylheptanoic fatty acid. Polymyxin B, by contrast, contained mostly the B1 subtype that has a more hydrophobic 6-methyloctanoic acid as its fatty acid moiety.

**Figure 6:**
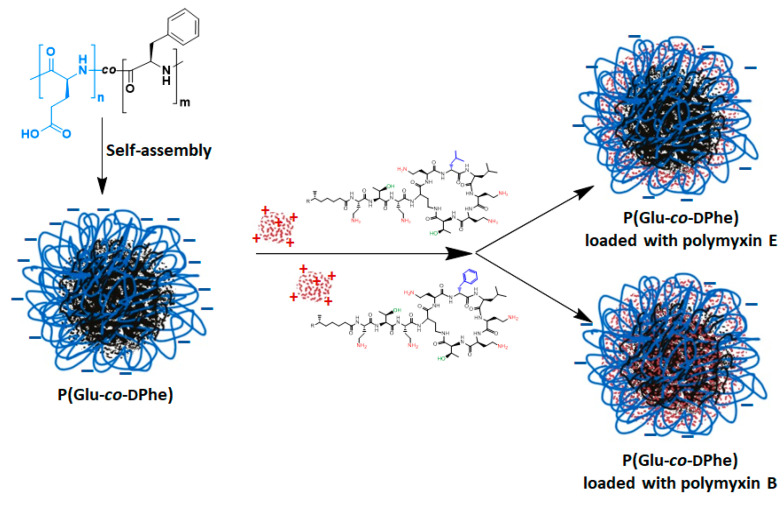
Scheme of polymyxin encapsulation into P (Glu-*co*-dPhe) nanoparticles.

Drug loading and release kinetics are crucial properties of a delivery system. Polymyxins were loaded into the polypeptide NPs by entrapping the drugs in the NPs immediately after their redispersion. The scheme of polymyxin loading into P (Glu-*co*-dPhe) NPs is presented in Figure 6.

**Figure 7:**
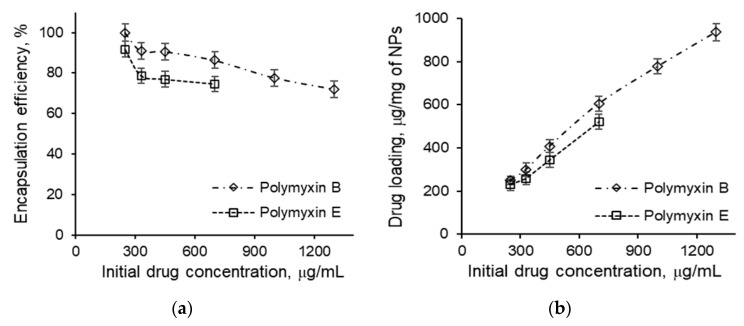
Dependencies of encapsulation efficacy (**a**) and drug loading (**b**) for polymyxins B and E on initial drug concentration applied for loading.

The encapsulation efficacy (EE) and loading capacity (LC) values were determined as a function of drug concentration (Figure 7). An increase in the initial drug concentration led to a different behavior of polymyxins B and E. For polymyxin B, a variation in the initial concentration from 0.25 to 1.30 mg/mL reduced the EE from 100% to 72% without aggregation of NPs. By contrast, for polymyxin E, the EE was slightly lower than for polymyxin B. In particular, an increase in the initial polymyxin E concentration from 0.25 to 0.7 mg/mL was accompanied with a decline in the EE from 92% to 74%. An increase in the initial drug concentration higher than 1.3 mg/mL for polymyxin B and higher than 0.7 mg/mL for polymyxin E resulted in aggregation of the NPs. Thus, the maximal loading of polymyxin B was 910 ± 30 µg/mg of NPs, whereas this value for polymyxin E was only 520 ± 20 µg/mg. 

The differences observed in loading can be related to the higher hydrophobicity of polymyxin B1, which dominated the commercial polymyxin B sample. By contrast, polymyxin E2 was the main subtype in the commercial polymyxin E sample. As a result, the hydrophobic interactions between the peptide drug and the polymer were much more pronounced for the polymyxin B sample. In addition, π–π interactions between the phenylalanine residues in the polypeptide and polymyxin B can be also a force that drives greater loading of the antibiotic into the hydrophobic core of the NPs. In turn, the more hydrophilic polymyxin E seemed to be localized predominantly on the surface of the NP or close to it. Thus, surface charge neutralization proceeds much faster and the aggregation of NPs occurs at lower initial concentrations for polymyxin E than for polymyxin B. 

The measurement of the hydrodynamic diameter and ζ-potential for NPs loaded with 420 µg/mg of polymyxin B and 355 µg/mg of polymyxin E showed a decrease for both *D_H_* and ζ-potential in comparison with initial values (Table 2). In particular, the *D_H_* values were 147 nm (PDI 0.17) for polymyxin B and 136 nm (PDI 0.23) for polymyxin E, while the ζ-potential decreased to −36 mV for both systems. These results indirectly support the assumptions made about the differences in polymyxin B and E entrapment. The slightly higher compaction of NPs in case of polymyxin E and the same charge decrease as for polymyxin B at lower loading may again indicate a localization of polymyxin E close to the NP surface.

In general, the determined values for the EE are quite high in comparison with results published for other NPs. For example, the reported EE for polymyxin E into different liposomes ranged from 45% to 67% [24,25,47]. For polymeric carriers, an EE of 47% was reported for alginate/chitosan microparticles [31,32]. 

### 3.5. Polymyxin Release 

**Figure 8:**
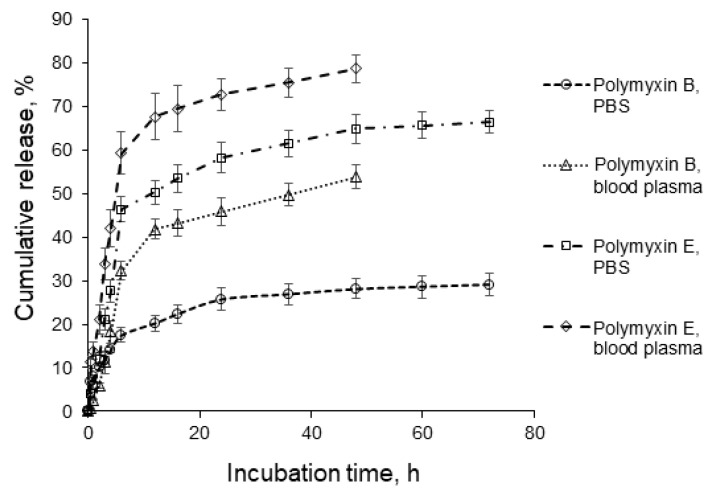
Cumulative release profiles of polymyxins B and E into buffer solution (0.01 M PBS, pH 7.4) and into human blood plasma.

The cumulative release of both antibiotics at 37 °C was studied in 0.01 M PBS (pH 7.4) and human blood plasma. Figure 8, shows that the rate of release was higher for polymyxin E than for polymyxin B. In buffer solution, 73% of the polymyxin E but only 30% of the polymyxin B was released after 72 h. By comparison, Fu et al. reported a release of more than 80% of the polymyxin B from chitosan-modified liposomes in PBS at 37 °C over 12 h and complete drug release by 24 h [48]. 

Wallace et al. reported a 46–56% release of polymyxin E in 2 h, depending on the initial drug loading, under the same model conditions [24]. A similar fast release was also observed by Coppi et al. The release of polymyxin B from alginate/chitosan polyplexes into a buffer solution (pH 7.4) was 50–60% after 2 h [32]. In the present study, the release of polymyxins B and E in PBS was 12% and 21%, respectively, after 3 h for the polypeptide NPs. The reason for this slower release of polymyxins from P(Glu-*co*-dPhe) NPs may be the multiple electrostatic and hydrophobic interactions involved in the peptide entrapment. The difference in the rate of polymyxin B and E release can also be related to these entrapment features.

The obtained experimental results have been supported with theoretical calculations. Modeling of the interactions between polymyxin B/colistin and pentapeptide Glu-Glu-Phe-Glu-Glu, as simple segment of random polypeptide, by molecular docking allowed the calculation of binding energy. It was found that binding energies for polymyxin B-pentapeptide and colistin-pentapeptide were calculated as 8.9 and 8.5 kJ/mol, respectively. The obtained result testified the slightly higher binding energy, which means stronger interaction of polymyxin B with pentapeptide. Taking into account that the polypeptide chain is much longer than pentapeptide fragment used for calculations, one can expect the total stronger interaction between polymyxin B and P(Glu-*co*-dPhe) NPs in comparison with polymyxin E.

The release into a biological fluid showed a similar trend to the release into buffer. However, for both antibiotics, the rate of release was higher into human blood plasma. About 80% of the polymyxin E and 50% of the polymyxin B was released after 48 h. The higher release in biological fluid is associated with the competitive interaction of the polypeptide with plasma proteins. This influence of protein present in the medium on the peptide release is already known. For instance, a faster release of polymyxin E from liposomes in diluted bovine serum in comparison with buffer solution has been reported elsewhere [25]. 

The effects of diffusion, dissolution, and polymer relaxation factors on the release of polymyxins B and E from P(Glu-*co*-dPhe) NPs were evaluated by analyzing the release data using a number of mathematical drug dissolution kinetic models (Table 4 and Figure S7).

Polymyxins B and E showed different approximation constants and dissolution constants, indicating that their release mechanisms are different. The surrounding medium also clearly has an effect on the release process. The release of polymyxin B into PBS was barely approximated by the models, which are based on dissolution control of the drug release process (zero-order and Hixon-Crowell models). The unsatisfactory correlation of the release profile with the Hopfenberg model indicates that matrix erosion is not a factor that governs the release process in this case. The diffusion-based Higuchi model had a better fit with the initial stage of polymyxin B release into PBS. The corresponding *n* parameter determined from Korsmeyer-Peppas model within first 6 h of release was equal to 0.43 (Table 4), revealing that Fick’s diffusion from spherical matrix is the main mechanism for the release process. 

Notably, placement of polymyxin B-loaded P(Glu-*co*-dPhe) NPs into human blood plasma resulted in release data that were better approximated by the dissolution and erosion-based models. The *n* parameter from the Korsmeyer-Peppas model was 1.49 (Table 4), revealing the anomalous Super Case II diffusion mechanism of drug release, which is a complex mechanism involving a combination of diffusion, relaxation, and erosion. This mechanism is associated with release from the spherical systems with a vitreous nucleus covered by an outer gel layer. Polymer container crazing is also an important factor associated with the Super Case II release mechanism [49]. 

The initial stage of polymyxin E release from P(Glu-*co*-dPhe) NPs, both in PBS and blood plasma, was in a good agreement with the models based on dissolution and erosion control over release process (Hixon-Crowell and Hopfenberg models). The Korsmeyer-Peppas model indicates a Super Case II release mechanism (*n* = 1.17) into PBS and Case II transport (*n* = 0.81) for release into blood plasma. The Case II release mechanism is associated with an equal involvement of diffusion and polymer relaxation in the drug release process. 

The described peculiarities are in line with the supposed differences in the hydrophobicity of polymyxin B and E. In the case of polymyxin B, it appears to enter into the hydrophobic core of the P(Glu-*co*-dPhe) NPs. Thus, the release in PBS is limited by the slow diffusion back out from the hydrophobic core. The release in blood plasma is affected by albumins, which can interact with particles and destabilize the hydrophobic core structure. Obviously, this type of interaction would facilitate the dissolution of polymyxin B from the NPs. The lower hydrophobicity of polymyxin E results in its preferential arrangement on the surface of the NPs, so its release is governed more by dissolution than by diffusion. This dissolution is more rapid in human blood plasma than in PBS, so the dissolution constant values are higher (Table 4).

One important point to note is that the best fit of the full-time release profile was observed for the Gompertz, Weibull, and Peppas-Sahlin two-parameter models (Table 4 and Figure S7). The Gompertz model is recognized as useful for comparing the release profiles of drugs having good solubility and an intermediate release rate, which is the case for polymyxin release from P (Glu-*co*-dPhe) NPs. The *β* parameter in the Gompertz model (Table 4) expresses the dissolution rate, which is the lowest for polymyxin B in PBS and highest for polymyxin E in human blood plasma. Interestingly, the dissolution rate for polymyxin E in PBS is two times higher than that for polymyxin B in the same medium, thereby confirming the surface arrangement of polymyxin E within the NPs.

The Weibull model is not associated with any specific mechanism of release, but it allows evaluation of the time dependence parameter *α* and the dissolution curve progression parameter *β*. The *α* parameter was the same for polymyxin B in either PBS or blood plasma, but it differed for polymyxin E release into the two different media, with the minimum time for the release process occurring in human blood plasma. The *β* values are below 1, which indicates a high initial slope of the exponential curve. This seems to be rational for a drug with a high solubility in water.

The Peppas-Sahlin model allowed comparison of the contribution of Fickian diffusion (f) and polymer relaxation (R) to the release process (Figure S8). The performed calculations revealed that Fickian diffusion is more important at the initial stage, while polymer relaxation becomes more important factor later in the release. The release of polymyxins B and E into PBS was also mostly associated with Fickian diffusion, while release of peptides into human blood plasma was strongly polymer relaxation. As already mentioned, the possible reason for this is an interaction of particles with albumins and other macromolecular serum components. 

### 3.6. Antibacterial Activity

**Figure 9:**
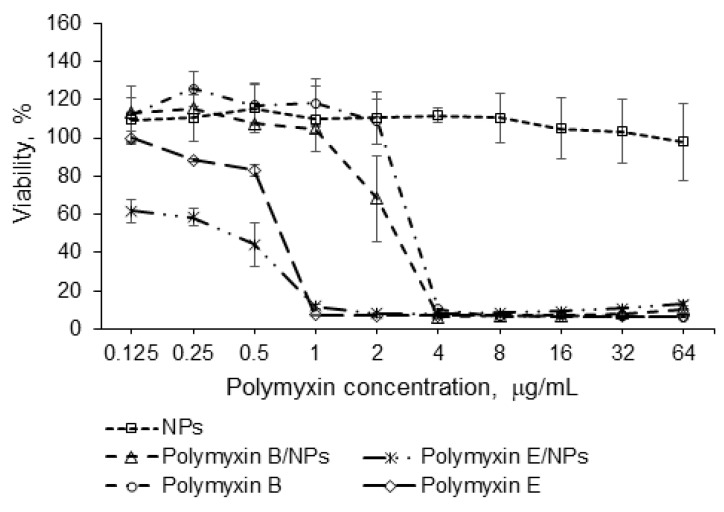
Antibacterial activity of free polymyxins B and E and their nanoformulations against *Pseudomonas aeruginosa.* The data are given as mean ± SD. The differences between all groups were statistically significant (*p* < 0.05) except concentrations of 2 and 4 μg/mL for encapsulated and free polymyxin E (*p* > 0.05).

The antibacterial activity of the prepared formulations and free drugs was evaluated by determining the MIC using a *P. aeruginosa* culture at a concentration of 10^7^ CFU/mL. Empty P(Glu-*co*-dPhe) NPs were also tested to determine what inhibitory effect, if any, they might have on the selected bacterial strain. The results presented in Figure 9 show that unloaded negatively charged polypeptide NPs did not affect the growth of *P. aeruginosa*. At the same time, both polymyxin B and E nanoformulations demonstrated an evident antibacterial effect. The MIC of the encapsulated form of polymyxin B was 4 μg/mL, which coincided with the MIC of the free antibiotic. In turn, the MIC of the polymyxin E loaded into the polypeptide NPs was also the same as the MIC detected for the free drug, namely, 1 μg/mL. However, the survival rate of a microorganism culture was lower for polymyxin E nanoformulation than for free antibiotic at concentrations of 0.125–0.5 μg/mL. 

According to the literature data, the MICs for different nanoformulations of polymyxins can be higher or the same as that of the free drug. For example, Omri et al. detected a better antimicrobial effect against a *P. aeruginosa* strain with a liposomal polymyxin B than with the free drug [50]. Fu et al. observed that the MIC for chitosan-modified polymyxin B-loaded liposomes was 8 µg/mL, while that one for free polymyxin B was 32 µg/mL [48]. When liposomes were used as delivery systems, the enhanced antimicrobial activity was usually explained by the fusion interaction of the phospholipid membrane of the liposomes with the bacterial cells [51]. At the same time, liposomes demonstrate fast release kinetics for polymyxins and low in vivo stability compared with polymer NPs. In turn, for NPs of other natures, the MIC of a free polymyxin and its nanoformulations against *P. aeruginosa* strain was reported to be the same. For instance, the MIC of poly(butyl cyanoacrylate) NPs loaded with polymyxin B was 2 μg/mL. The similarity of the MIC for colistimethate loaded into lipid NPs and that of free drug was also established by Moreno-Sastre et al [26]. In both cases, the MIC was 16 μg/mL.

In summary, the P(Glu-*co*-dPhe) NP delivery systems demonstrated good preservation of the biological activity of polymyxins as well as high stability in biological media, appropriate drug release kinetics, low cytotoxicity, and reduced uptake by macrophages.

## 4. Conclusions

In this work, P(Glu-*co*-dPhe) was synthesized and carefully characterized in regards to the molecular weight, polydispersity, and composition. Being amphiphilic, P(Glu-*co*-dPhe) had a tendency to self-assemble into spherical NPs that were stable and noncytotoxic, and demonstrated a low rate of uptake by macrophages when compared with standard PLA and PLA-*b*-PEG NPs. The uptake by macrophages was further reduced by modification of P(Glu-*co*-dPhe) NPs with the “self”-peptide. This finding has important implications for consideration of nanomedicines under study for intravenous administration. 

The encapsulation efficiency and drug loading were determined for both polymyxins B and E into P(Glu-*co*-dPhe) NPs. Polymyxin B showed a higher level of loading than one observed for polymyxin E. The release rate was in agreement with the loading features, as polymyxin E was released faster than polymyxin B in both a buffer solution and a complex biological fluid like human blood plasma. The release of polymyxins B and E into PBS is mostly associated with Fickian diffusion, while the release of peptides into human blood plasma is attributed to polymer relaxation. The prepared polymyxin formulations demonstrated MICs that were equal to those of the free antibiotics. In comparison with other reported polymyxin delivery systems, P(Glu-*co*-dPhe)-based polymyxin nanoformulations surpass some of them because of their complex physicochemical and biological properties. The developed nanoformulations seem to have potential for future in vivo testing.

## Figures and Tables

**Table 1:** Molecular weight characteristics and composition of P(Glu-*co*-dPhe) samples.

Sample	Glu NCA_0_/dPhe NCA_0_ (mol/mol)	SEC			SLS	HPLC
		*M_w_*	*M_n_*	*Ɖ*	*M_w_*	Glu/dPhe (mol/mol)
1	80/20	7800	6500	1.20	7000	75/25
2	86/14	8100	6600	1.23	8000	80/20

*Conditions: n*-hexylamine was used as the initiator; [Glu + dPhe]/[I] = 100; 4 wt.% monomer solution in THF was used for the synthesis; temperature and polymerization time were 25 °C and 48 h, respectively.

**Table 2:** Characteristics of nanoparticles based on P(Glu-*co*-dPhe).

Sample	Characteristics			
	*D_H_,* nm	PDI	ζ-Potential, mV	CCM, µg/mL
1	150 ± 19	0.22	−46.0 ± 0.9	–
2	162 ± 22	0.25	−48.4 ± 1.8	4.2

**Table 3:** Composition of commercial polymyxin preparations for drug loading in the study.

Sample	Subtype	m/z	Content, %
Polymyxin B	B1	602.3824	81.5 ± 0.6
	B2	595.3760	18.5 ± 0.6
Polymyxin E	E1	585.3890	31.1 ± 0.4
	E2	578.3808	68.9 ± 0.4

**Table 4:** Correlation coefficients and dissolution constants evaluated by fitting the polymyxin B and E release profiles into different media with mathematical models. The linearization curves are presented in Supplementary Materials (Figure S7).

Model	Polymyxin B		Polymyxin E	
	PBS	Human Blood Plasma	PBS	Human Blood Plasma
Zero-order *	R^2^ = 0.6401 K_zo_ = 3.44	R^2^ = 0.9471 K_zo_ = 4.75	R^2^ = **0.9885** K_zo_ = 7.30	R^2^ = **0.9740** K_zo_ = 10.37
First-order *	R^2^ = 0.6900 K_fo_ = 0.04	R^2^ = 0.9215 K_fo_ = 0.05	R^2^ = **0.9620** K_fo_ = 0.09	R^2^ = **0.9844** K_fo_ = 0.14
Higuchi *	R^2^ = **0.9793** K_H_ = 7.17	R^2^ = 0.7064 K_H_ = 8.88	R^2^ = 0.8012 K_H_ = 14.00	R^2^ = 0.9180 K_H_ = 20.57
Higuchi **	R^2^ = 0.8303 K_H_ = 4.98	R^2^ = 0.8968 K_H_ = 8.82	R^2^ = 0.8707 K_H_ = 11.29	R^2^ = 0.7938 K_H_ = 14.47
Korsmeyer-Peppas *	R^2^ = **0.9895** K_KP_ = 7.91 n = 0.43	R^2^ = **0.9970** K_KP_ = 2.26 n = 1.49	R^2^ = **0.9989** K_KP_ = 5.64 n = 1.17	R^2^ = **0.9915** K_KP_ = 13.66 n = 0.81
Hixon-Crowell *	R^2^ = 0.6741 K_HC_ = 0.01	R^2^ = 0.9302 K_HC_ = 0.02	R^2^ = **0.9720** K_HC_ = 0.03	R^2^ = **0.9863** K_HC_ = 0.04
Hopfenberg *	R^2^ = 0.6899 K_Hb_ = 0.01	R^2^ = **0.9935** K_Hb_ = 0.13	R^2^ = **0.9983** K_Hb_ = 0.12	R^2^ = **0.9863** K_Hb_ = 0.04
Hopfenberg **	R^2^ = 0.3805	R^2^ = 0.8804	R^2^ = 0.8483	R^2^ = 0.8714
Baker-Lonsdale **	R^2^ = 0.8638 K_BL_ = 0.001	R^2^ = 0.9105 K_Hb_ = 0.002	R^2^ = 0.9130 K_Hb_ = 0.003	R^2^ = 0.9121 K_Hb_ = 0.007
Weibull **	R^2^ = **0.9867** α = 10.8 β = 0.35	R^2^ = 0.9226 α = 10.8 β = 0.59	R^2^ = 0.9315 α = 6.8 β = 0.56	R^2^ = 0.9497 α = 4.3 β = 0.55
Gompertz ***	R^2^ = **0.9899** α = 2.5 β = 0.4	R^2^ = **0.9664** α = 3.1 β = 1.0	R^2^ = **0.9714** α = 2.5 β = 1.1	R^2^ = **0.9785** α = 1.9 β = 1.3
Peppas-Sahlin ***	R^2^ = **0.9967** K_1_ = 8.8 K_2_ = 0.7 m = 0.45	R^2^ = **0.9564** K_1_ = 7.1 K_2_ = 0.2 m = 0.75	R^2^ = **0.9584** K_1_ = 13.5 K_2_ = 0.7 m = 0.59	R^2^ = **0.9687** K_1_ = 20.7 K_2_ = 1.4 m = 0.59

* 6 h release approximation; ** 0–48 h release approximation; *** full curve approximation.

## Supplementary Materials

The following are available online at https://www.mdpi.com/1999-4923/12/9/868/s1, Figure S1: Molecular docking of interaction between polymyxin E and pentapeptides: (a) Glu**_5_** and (b) Glu**_2_**PheGlu**_2_**; Figure S2: Size exclusion chromatography (SEC) traces of P(Glu-*co*-dPhe) samples; Figure S3: NMR spectra of P(Glu (OBzl)-*co*-dPhe) (a) and P(Glu-*co*-dPhe) (b) in DMSO-d6; Table S1: Characteristics used for calculations of *M_w_* for synthesized copolymers by static light scattering; Figure S4: Dependence of conductivity on polymer concentration in solution: determination of critical micelle concentration (CMC) by conductometry; Table S2: Characteristics of poly(lactic acid) (PLA) and poly(lactic acid)-*b*-poly(ethylene glycol)-5000 (PLA-*b*-PEG) nanoparticles used as control for comparison phagocytosis rate; Figure S5: Stability of P(Glu-*co*-Phe) and P(Glu-*co*-dPhe) nanoparticles in the enzyme containing medium; Figure S6: Uptake rate of fluorescently-labeled P(Glu-*co*-dPhe) nanoparticles (NPs) and those modified with “self”-peptide by macrophages; Figure S7: Drug dissolution kinetics models and approximation curves; Figure S8: The dependence of drug dissolution mechanism with time.
