# Supplementary material for: Polypeptide Self-Assembled Nanoparticles as Delivery Systems for Polymyxins B and E
Source: Pharmaceutics. 2020 Sep 11;12(9):868. doi: 10.3390/pharmaceutics12090868 (PMC7558620; doi:10.3390/pharmaceutics12090868)
Supplement: Supplementary file 1 [file pharmaceutics-12-00868-s001.pdf]

Supplementary Materials: Polypeptide self-assembled nanoparticles as delivery systems for polymyxins B and E

Dmitrii Iudin, Natalia Zashikhina, Elena Demyanova, Viktor Korzhikov-Vlakh, Elena Shcherbakova, Roman Boroznjak, Irina Tarasenko, Natalya Zakharova, Antonina Lavrentieva, Yury Skorik and Evgenia Korzhikova-Vlakh

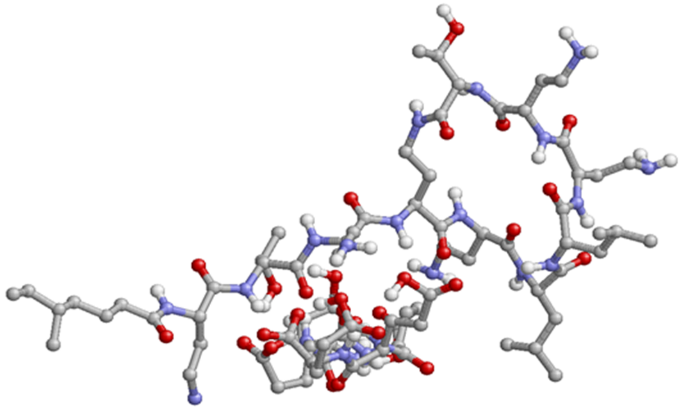

(a)

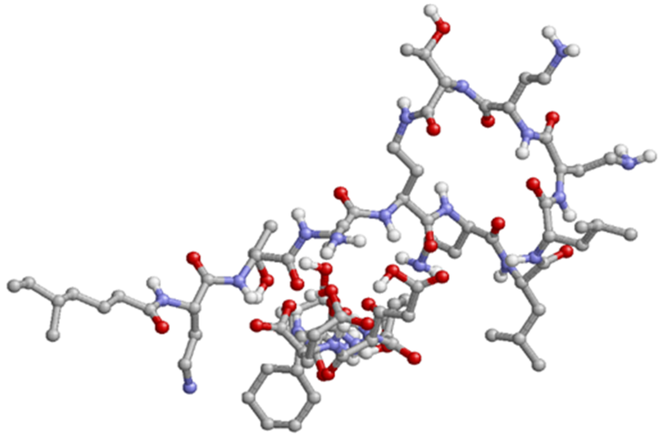

(b)

Figure S1. Molecular docking of interaction between polymyxin E and pentapeptides: *(a)* Glu_5; *(b)* $Glu_2PheGlu_2$.

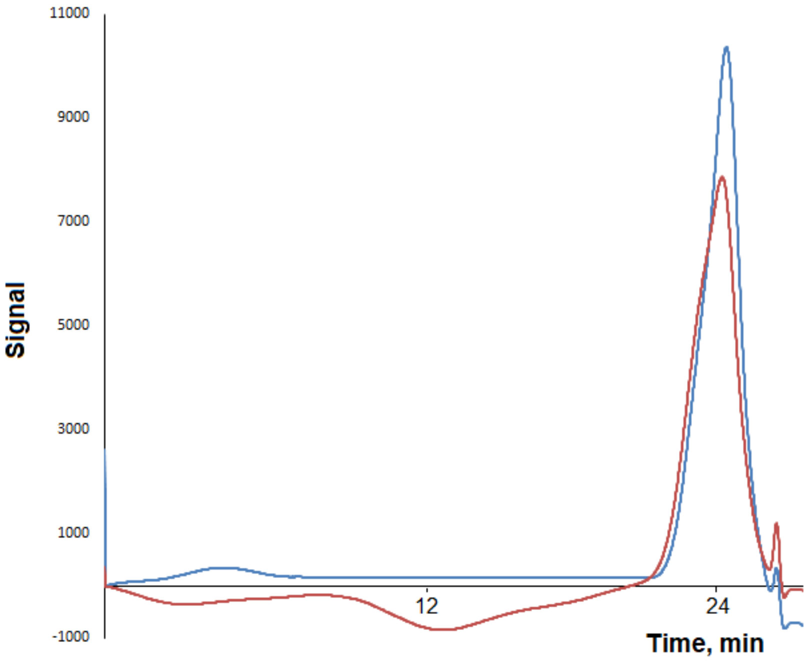

Figure S2. SEC traces of P(Glu-*co*-DPhe): blue line – sample 1; red line – sample 2.

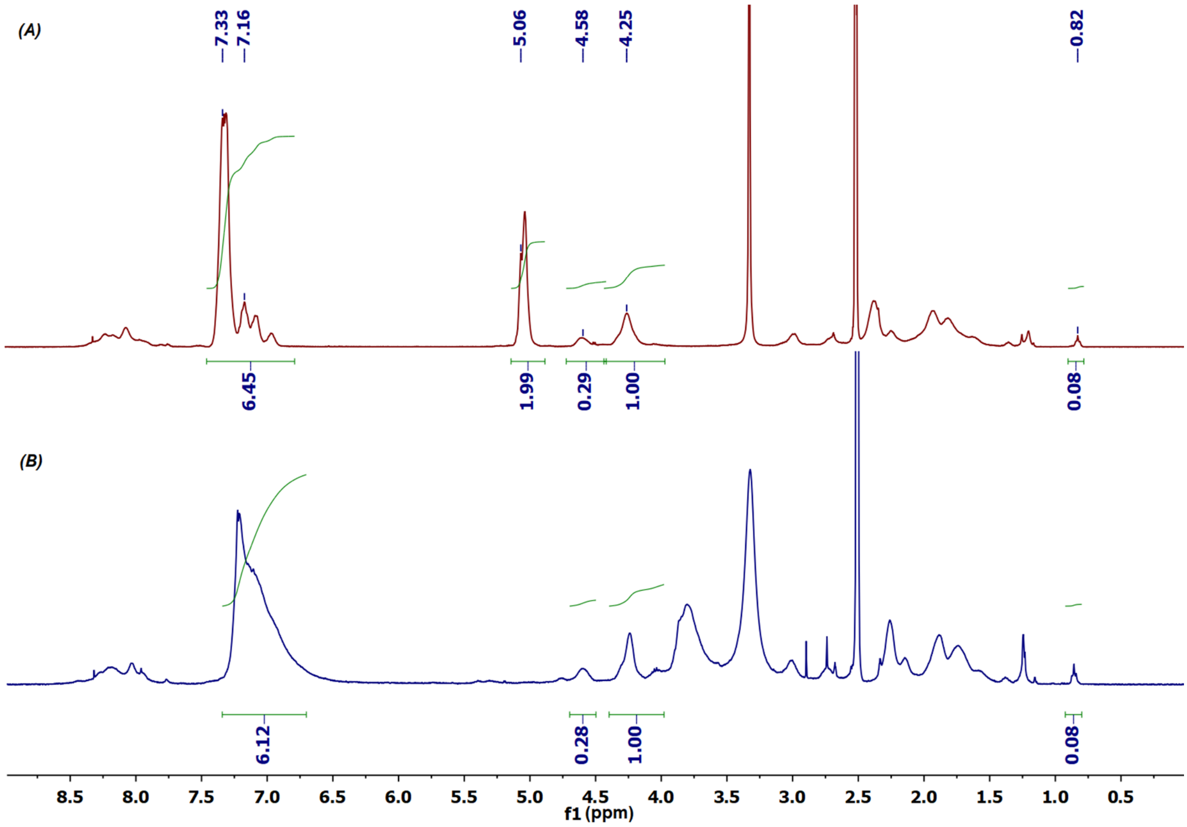

Figure S3. NMR spectra of P(Glu(OBzl)-*co*-DPhe) *(a)* and P(Glu-*co*-DPhe) *(b)* in DMSO-d6.

Table S1. Characteristics used for calculations of M_w for synthesized copolymers by static light scattering.

Sample	*dn*/*dc*, cm³/g	A_2, cm³·mol·g⁻²
1	0.1132	−8.56E−05
2	0.1171	−3.49E−04

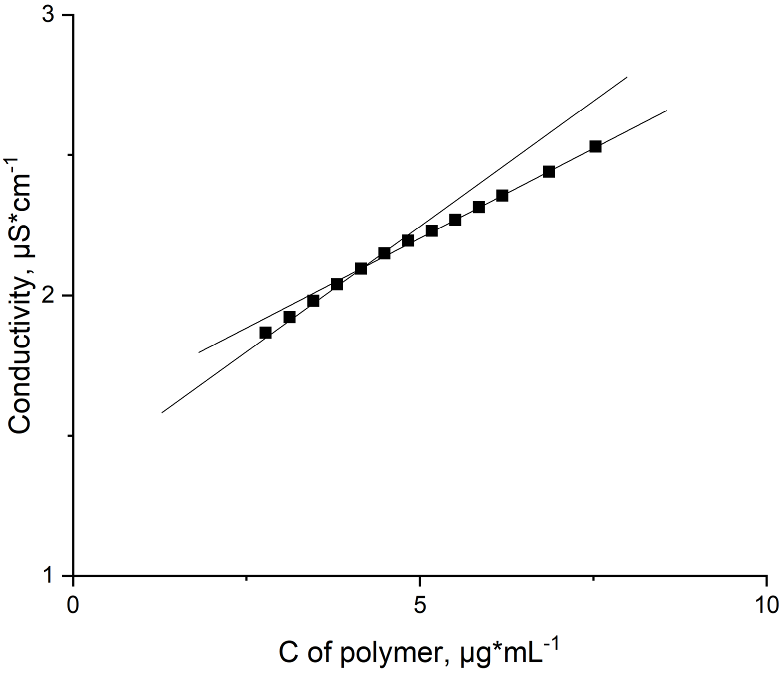

Figure S4. Dependence of conductivity on polymer concentration in solution: determination of critical micelle concentration (CMC) by conductometry.

Table S2. Characteristics of PLA and PLA-*b*-PEG nanoparticles used as control for comparison phagocytosis rate.

Nanoparticles	D_H, nm	ζ-potential, mV
PLA	92 ± 13	−8.1 ± 2.2
PLA-*b*-PEG	121 ± 16	−0.6 ± 1.1

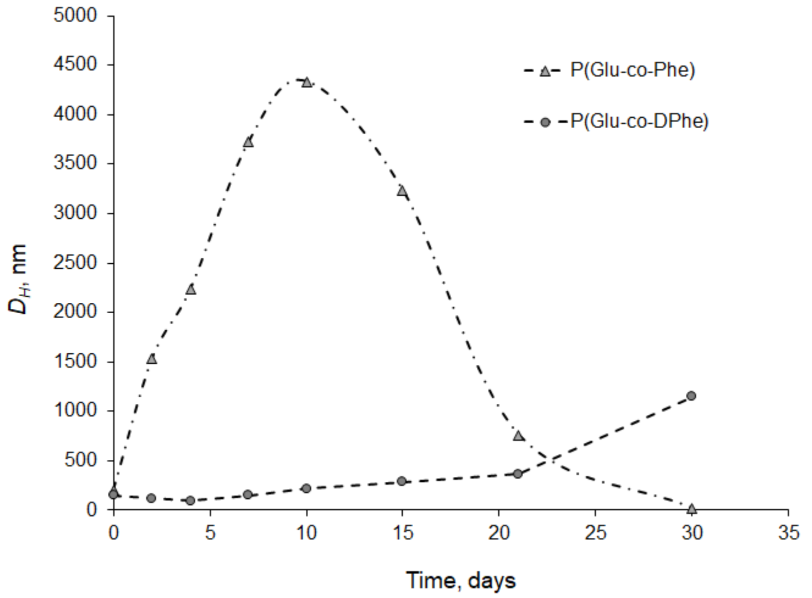

Figure S5. Stability of P(Glu-*co*-Phe) and P(Glu-*co*-DPhe) nanoparticles in the enzyme containing medium. *Conditions:* 0.01 M PBS containing papain; the concentration of nanoparticles was 1.0 mg/mL; the concentration of papain was 0.05 mg/mL; the suspension was incubated at 37 °C. In 15 days the medium was replaced with a fresh portion.

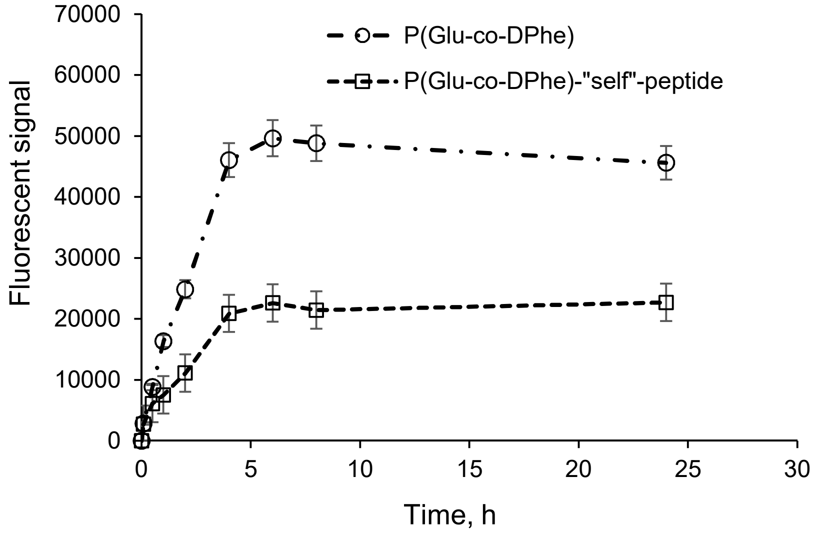

Figure S6. Uptake rate of fluorescently-labeled P(Glu-*co*-DPhe) NPs and these modified with "self"-peptide by macrophages. *Conditions:* J774A.1 cell line (mouse macrophages); mouse "self"-peptide: GNYTCEVTELTREGETIIELK; flow cytometry; concentration of NPs was 50 µg/mL; amount of bound "self-peptide" as 50 µg/mg of NPs; dansylcadaverine was used as a fluorescent label.

Zero-order $F=k_{zo}*t$

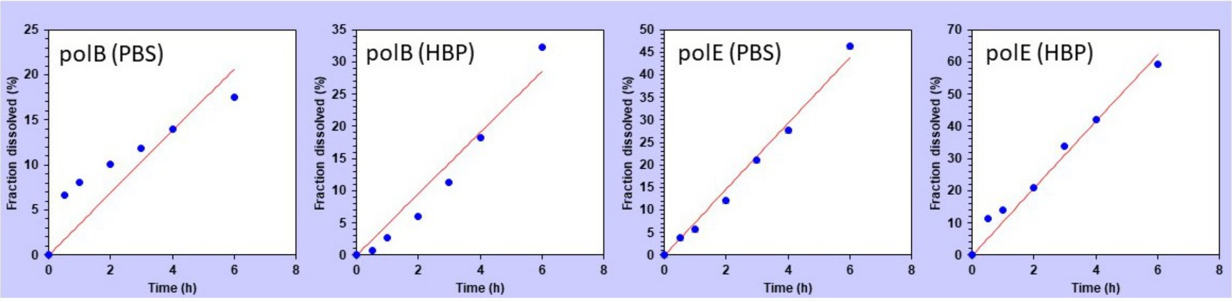

First-order $F=100*[1-Exp(-k_{fo}*t)]$

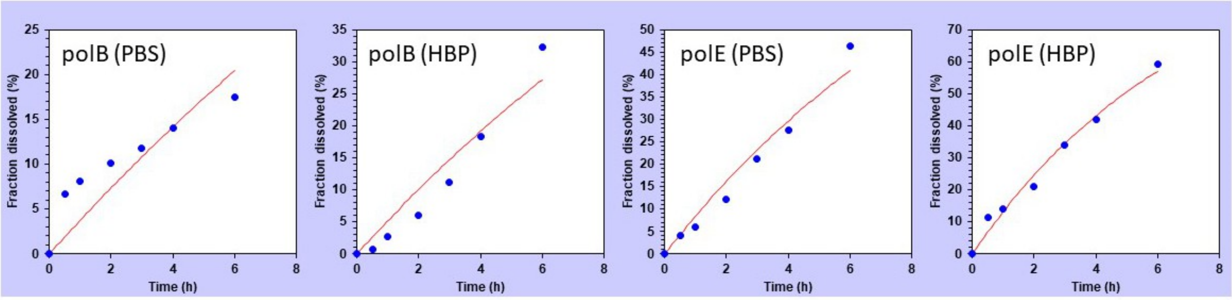

Higuchi $F=k_H*t^{0.5}$

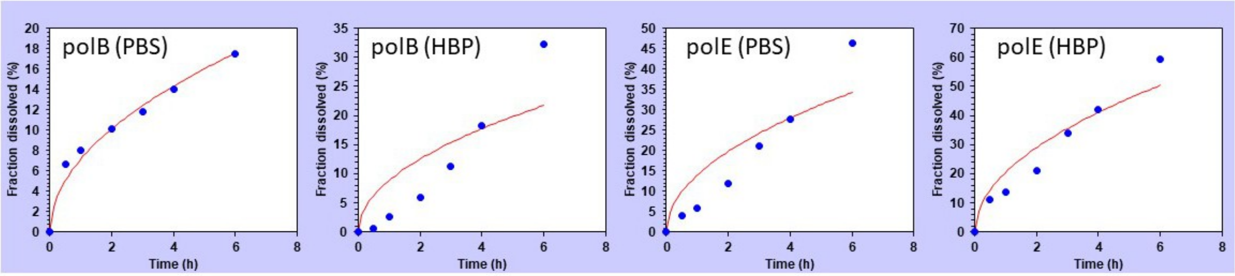

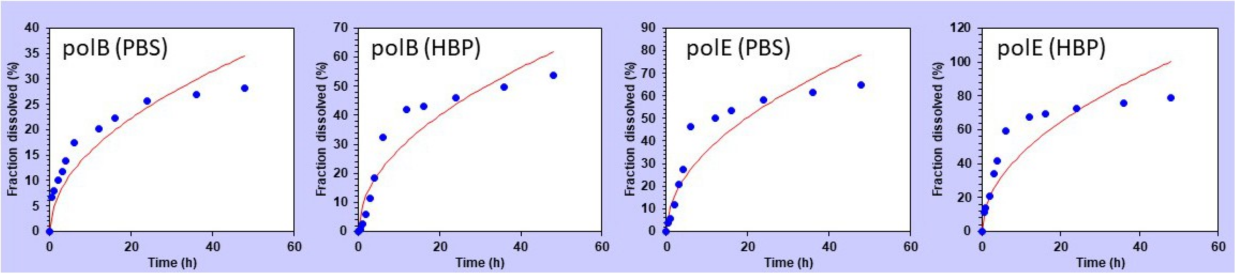

Korsmeyer-Peppas $F = k_{KP} * t^n$

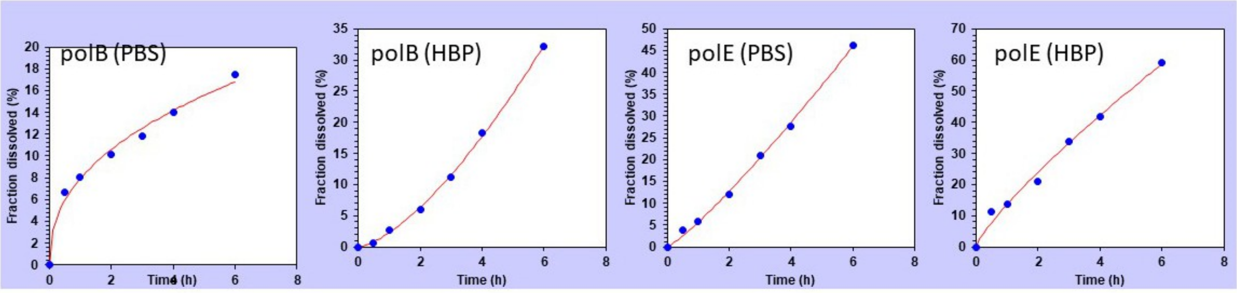

Hixson-Crowell $F = 100 * [1 - (1 - k_{HC} * t)^3]$

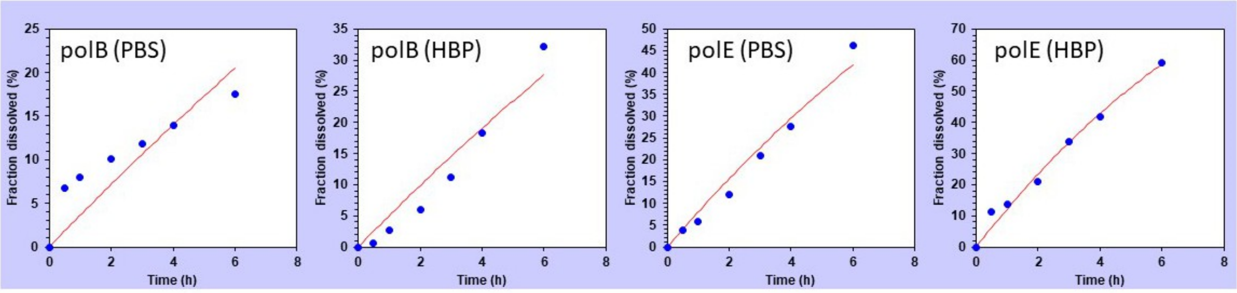

Hopfenberg $F = 100 * [1 - (1 - k_{HB} * t)^n]$

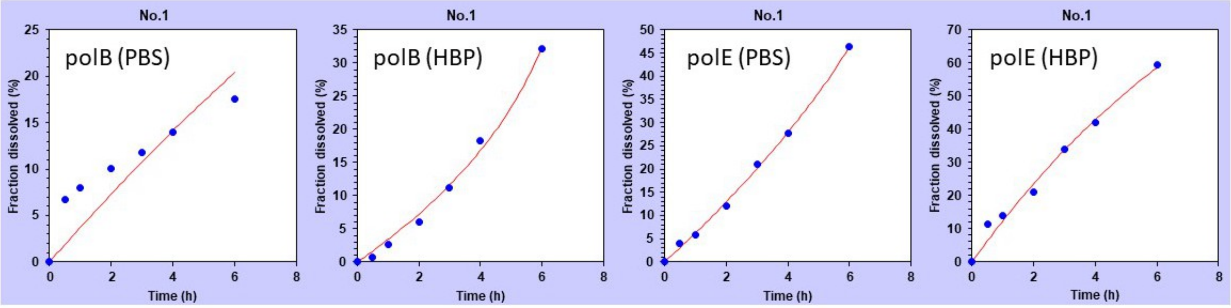

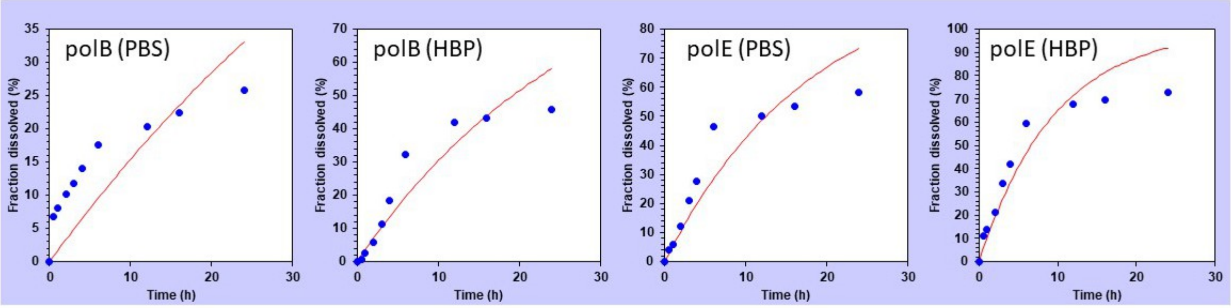

Weibull $F=100*\{1-Exp[-(t^{\beta})/\alpha]\}$

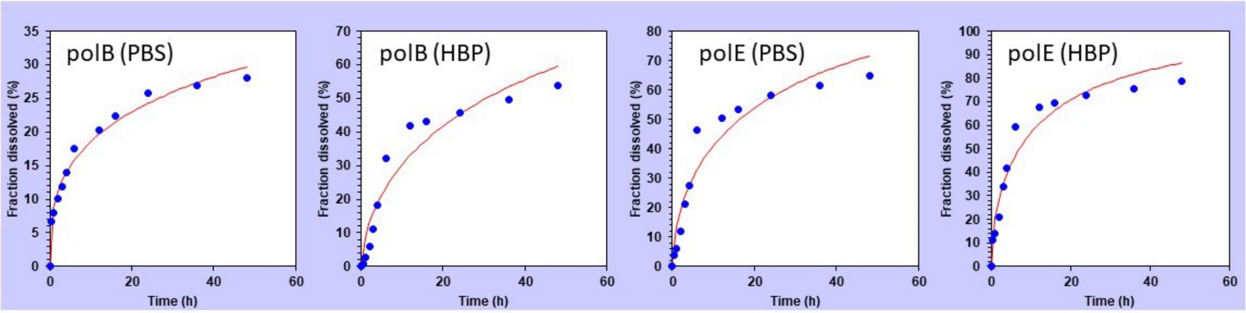

Gompertz $F=100*Exp\{-\alpha*Exp[-\beta*log(t)]\}$

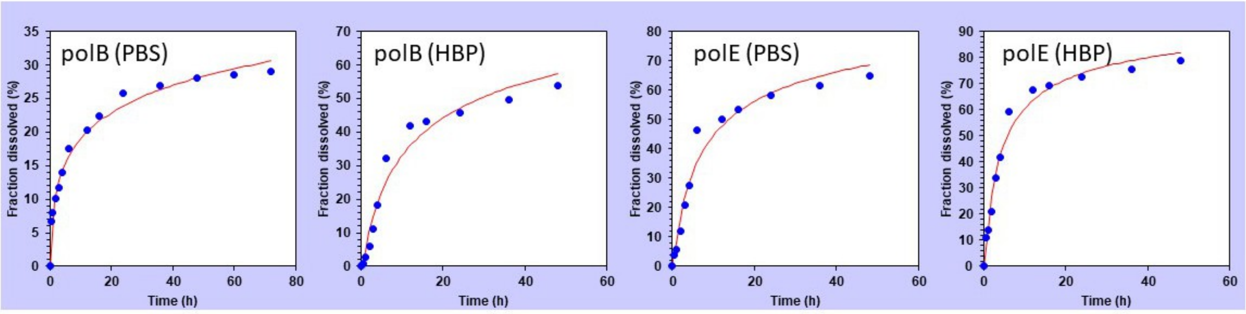

Peppas-Sahlin $F=K_1*t^m+K_2*t^{(2*m)}$

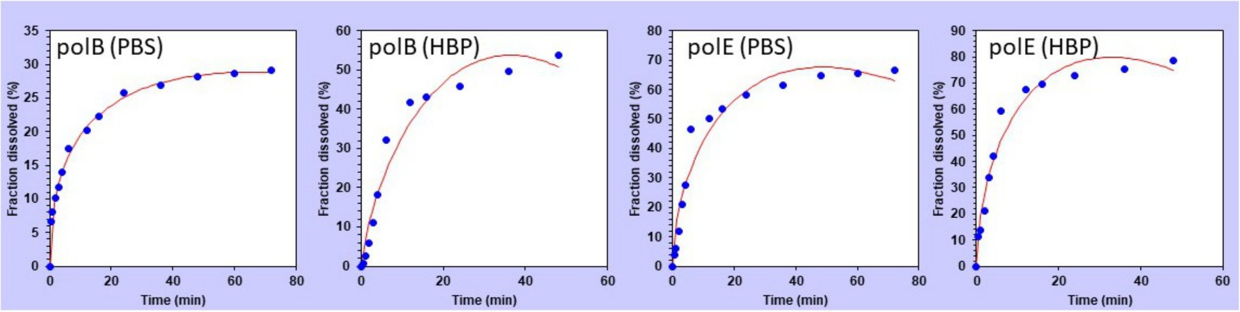

Baker-Lonsdale $3/2*[1-(1-F/100)^{(2/3)}]-F/100=k_{BL}*t$

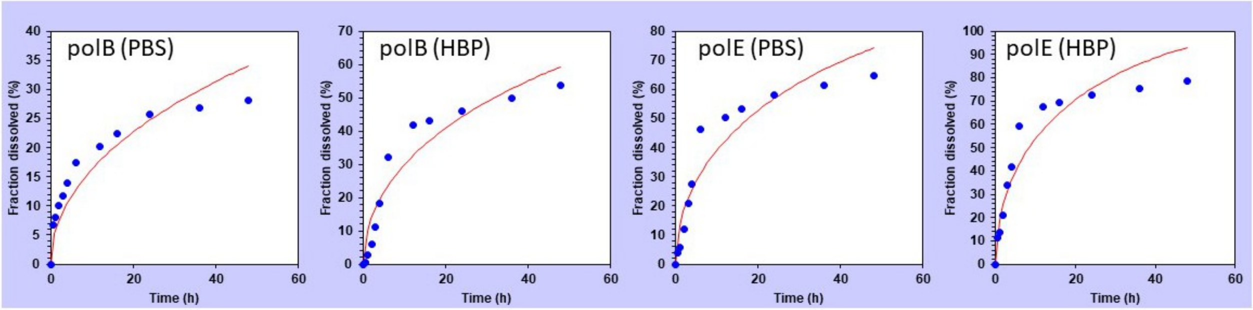

Figure S7. Drug dissolution kinetics models and approximation curves.
Abbreviations: PolE: polymyxin E; PolB: polymyxin B; PBS: 0.01 M sodium phosphate buffer, containing 0.15 mol/L NaCl; HBS: human blood plasma.

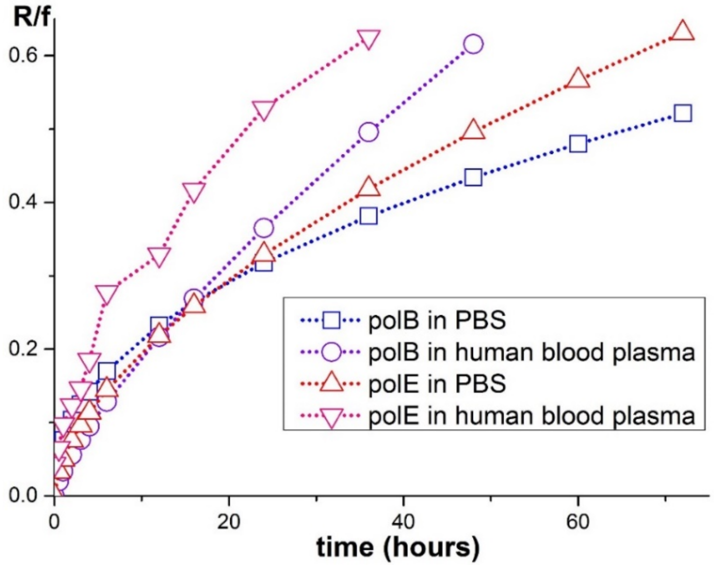

Figure S8. The dependence of drug dissolution mechanism with time. $\boldsymbol{R/f}$ is the ratio of polymer relaxation (R) to Fick's diffusion (f) as the driving mechanisms of drug release for the system. At definite time $\boldsymbol{R/f}$ was calculated as follows: $\frac{R}{f} = \frac{K_2 \times t^m}{K_1}$, were K_1, K_2 and m are parameters determined by approximation of release data with Peppas-Sahlin model and t is time. *Abbreviations:* PolE: polymyxin E; PolB: polymyxin B; PBS: 0.01 M sodium phosphate buffer, containing 0.15 mol/L NaCl; HBS: human blood plasma.
